# The 2021 Genetics Society of America Medal: Douglas Koshland

**DOI:** 10.1093/genetics/iyab232

**Published:** 2022-02-13

**Authors:** Douglas Koshland

**Affiliations:** Department of Molecular and Cell Biology, University of California, Berkeley, Berkeley, CA 94720-3202, USA

## Abstract

The Genetics Society of America Medal honors an individual member of the Society for outstanding contributions to the field of genetics in the last 15 years. Genetics Society of America established the Medal in 1981 to recognize members who exemplify the ingenuity of the Genetics Society of America membership through elegant and highly meaningful contributions to modern genetics. The 2021 Genetics Society of America Medal has been awarded to Douglas Koshland of the University of California, Berkeley. His advances in chromosome biology have not only illuminated fundamental features of the structure of chromosomes but also provided tools for many others to use.

**Figure:**
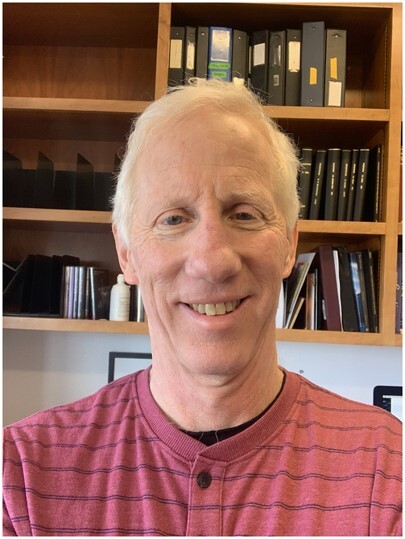


As the old saying goes, there is a thin line between love and hate. I hated genetics! But then I fell in love with it. The hate began as an undergraduate while I was in the process of deciding whether to major in biology or chemistry at Haverford College. Haverford had a fantastic biology department—one of the few in small colleges that taught and practiced molecular biology at that time. The introductory biology course included a section on genetics. For reasons baffling to me now, I took an extreme dislike for genetics and biology, becoming a chemistry major instead.

However, one of the remarkable features of Haverford was that all courses focused on learning critical abstract concepts rather than just information. So, unbeknownst to me, my mind was being prepared to learn the beautiful abstraction of genetics. I just needed to give it a second chance. That chance came when I started graduate school at Massachusetts Institute of Technology. Because of my chemistry background, they pinned me as a potential biophysicist. However, in their hubris, they made the entire entering graduate class take many of their science undergraduate courses, including genetics. The genetics course, taught by David Botstein, electrified me, marking the beginning of a long and beautiful relationship with genetics.

I joined David's laboratory for my thesis work, and I quickly learned that genetics was beautiful not only in abstraction but also in practice. It was a potent tool to interrogate the chemistry of living cells and, most importantly, to assess its biological significance. This revelation came from my studies of the secretion of beta-lactamase. At the time, elegant in vitro biochemical studies from the laboratory of Gunter Blobel had led him to postulate that protein secretion occurred by threading the emerging nascent polypeptide chain through a pore. This model quickly became the accepted mechanism of protein secretion, particularly when its in vivo relevance was supported by genetic experiments of the Silhavy and Beckwith laboratories. Blobel's discovery earned him the Nobel Prize. However, through my study of wild-type and unusual mutants of beta-lactamase, I discovered cells that were cleverer than we (the field) had thought: they could also secrete proteins like beta-lactamase long after they were finished being translated. To say that this conclusion was an unwelcome surprise to the field is an understatement.

This work taught me three important lessons. First, if my goal was to understand the chemistry of living cells and organisms, then genetic manipulation of that chemistry in vivo was critical to assess its biological significance. Second, wonderfully unexpected phenotypes of mutants would likely always humble me by revealing my inability to imagine the diversity of molecular biology in living cells honed by millions of years of evolution. Third, sticking by a surprising conclusion from a mutant phenotype requires a rigorous interpretation of that phenotype and proper controls. Fortunately, I have been taught by master geneticists first in graduate school (Fred Winston, Marian Carlson, Tom Petes, Mark Rose, and David Botstein) and throughout my career (Lee Hartwell, Barbara Meyers, Allan Spradling, and Jasper Rine). I came away from graduate school feeling unleashed (some would argue to my detriment). I could use genetic strategies to study any topic of my choosing, confident that those studies would yield important and often controversial biological insights that would fill me with satisfaction and wonder.

I chose my first topic by serendipity. In a graduate student journal club, Iva Greenwald presented a paper by Jonathan Hodgkin that used genetics to interrogate chromosome segregation in worms. That journal club led me to my two postdoctoral fellowships, first with Lee Hartwell and then with Marc Kirschner—two pioneers in cell division and chromosome segregation. Lee and Marc inspired me in many ways and gave me the courage to develop new assays for studying chromosome segregation. In Lee's laboratory, I developed novel assays with yeast minichromosomes (along with Phil Hieter, then in Ron Davis’s laboratory) to measure the fidelity of their replication and transmission, kinetochore function, and the role of DNA catenation in sister chromatid cohesion. In Marc's laboratory, I used fluorescent probes to follow the movement of isolated Chinese Hamster Ovary chromosomes on depolymerizing bovine microtubules. I learned that creating assays with unusual substrates gave me access to unique opportunities for interrogation. Not surprisingly, developing new assays has been and continues to be a priority for my laboratory.

Emboldened by my training in Lee and Marc's laboratory, I made the decision to start my own laboratory using budding yeast to address the daunting but fascinating questions about higher-order chromosome structure and function. Although the powerful methods of yeast genetics were well established, there was a slight hitch. Few people believed that yeast chromosome structure was anything like that found in larger eukaryotes because their mitotic chromosomes were not visible by conventional stains. However, this did not make sense to me—why should chromosomes be any different when so many other cell biological processes had been shown to be conserved between yeast and man? We performed a genetic screen to identify mutants that perturbed minichromosome transmission as assayed by the loss of a genetic marker, believing that minichromosomes would be hypersensitive sentinels of mutations also affecting natural chromosomes. This screen identified the SMC (Structural Maintenance of Chromosomes) genes, whose presence in bacteria, worms (Meyer laboratory) and frogs (Hirano and Mithison) suggested the existence of a ubiquitous and heretofore unknown conserved process important for chromosome transmission.

What were these processes? Well, at the same time as discovery of the SMC genes, we were developing fluorescent in situ hybridization (FISH) as a tool to assess yeast chromosome structure. This assay revealed the presence of sister chromatid cohesion and condensation, establishing budding yeast as a model for chromosome structure. We used our FISH assay to isolate mutants defective for sister chromatid cohesion and condensation. Lo and behold, some of the mutations that altered cohesion and condensation lay in the very same SMC genes we had discovered from our earlier screen. The study of our other mutants led to the identification of SMC-associated proteins. This work, alongside studies from the Nasmyth laboratory, Hirano and Mitchison, and the Meyer laboratories, all led to the discovery of a remarkably small number of SMC complexes that orchestrate higher-order chromosome structure in eukaryotic cells. Subsequent studies in bacteria revealed a similar function. We and others leveraged the SMC complexes and these assays to identify key cell cycle regulators and regulators of chromosome structure. Studies in humans have revealed that one SMC complex, called cohesin, has significant biomedical relevance. Cohesin defects are linked to 30% of cancers and many congenital disabilities.

In the past 15 years, our studies of cohesin have generated lots of surprises and fun. These unexpected outcomes have stemmed from our willingness to leave the comfort zone of only pursuing the phenotypes of null alleles, which are an important but limited tool in interrogating the function of a gene or process.

For example, our studies from 2012 to 2018 of hypomorphic and suppressor alleles of cohesin subunits suggested that cohesin was much more biochemically and structurally complex than the textbook cartoons depicted. By analyzing our mutants with novel genetic and molecular assays, we concluded that cohesin has multiple DNA-binding activities, robust ATPase activity after binding to DNA, and and unusual structural featrues such as self oligomerization and large conformational changes. The presence of these activities foreshadowed the remarkable discovery in 2018 that cohesin and other SMC complexes could extrude DNA loops as well as tether DNA regions in vitro and in vivo. We are currently using these mutants as a unique resource to dissect many of the unanswered molecular questions: what is the mechanism of looping and tethering? How do complexes know to loop or tether? How can looping activity be controlled spatially? In addition, determining the biological function of SMC looping, distinct from its already established importance in tethering, remains a huge challenge to this field. Genetics will be the only solution. Genetics will be the only solution.

Perhaps feeling too empowered by genetic strategies and new assays, I have given in to my promiscuous scientific interests. I have convinced my relatively small laboratory to investigate many biological processes in addition to higher-order chromosome structure, including DNA replication, chromosome movement, spindle orientation, genome evolution, genome instability, DNA–RNA hybrids, and stress biology. Like our studies of cohesin, these studies were also enhanced by studying nonnull alleles.

Our interest in chromosomes naturally led to an interest in genome instability, which is a feature of evolution, cancer cells, and congenital disabilities. DNA–RNA hybrids form by the hybridization of nuclear transcripts with the chromosomal DNA, displacing a single stranded loop of DNA, and are potentially a significant contributor to genome instability. Hybrids cause DNA damage and gross chromosomal rearrangements, but how and where in the genome this occurred was unclear. We developed new assays that allowed us to identify at high resolution the genomic sites of hybrid formation and, for the first time in any organism, the sites of hybrid-induced DNA damage. We showed that mutants with persistent hybrids induced DNA damage with unusual structures, impacting tens of kilobases. Suppressors of this phenotype revealed a major new concept in hybrid-induced genome instability; hybrids induce gross chromosomal rearrangements not only by generating DNA damage but also by making unusual damage that can only be repaired by error-prone pathways.

We also tinkered with using yeast to study the impact of repetitive DNA on genome instability and genome evolution. This work spurred an interest in studying natural traits of yeast. But which one? Well, for over a century, model systems have been chosen for their extreme properties to amplify and thereby enhance the study of basic cell biological processes (fast cell division, shattering of genomes, amplified protein secretion, etc.). So, the extreme desiccation tolerance of yeast seemed like an intriguing window to study the stresses of, and responses to, water loss.

Using budding yeast as a model, we developed high-throughput assays to screen for desiccation-sensitive yeast. Surprisingly, we discovered that high levels of the sugar trehalose and the protein Hsp12 (a member of the hydrophilin protein family) are necessary and sufficient for desiccation tolerance. They act, at least in part, by limiting in vivo protein aggregation and loss of membrane integrity. Furthermore, we showed that a subset of hydrophilins from animals could promote desiccation tolerance in yeast. These studies suggested that hydrophilins may be a novel class of uncharacterized stress factors. We are currently exploring their translational applications.

In closing, the word “we” has been used liberally throughout this essay. A more accurate term would be “they,” as the brains and heavy lifters in almost all the projects discussed were my outstanding laboratory members. Their joy and success in the practice of genetics have been amazing. Even with a small laboratory, there are too many contributors to mention by name here. While I have no favorite science children, I must mention Vincent Guacci, one of my earliest science children who developed FISH for yeast as a postdoc and then became my colleague of the past 25 years. He has been invaluable to our laboratory's success, co-directing many projects and training many laboratory members.

